# Could Perfusion and Pleth Variability Index Be Useful in Managing Treatment and Morbidity in Patients with Gastrointestinal Bleeding?

**DOI:** 10.3390/jcm15145561

**Published:** 2026-07-15

**Authors:** Halil Atasoy, Isil Muge Karabacak, Caglayan Keklikkiran, Aziz Gumus, Kamil Konur

**Affiliations:** 1 Department of Gastroenterology, Faculty of Medicine, Recep Tayyip Erdogan University, 53020 Rize, Türkiye; halil.atasoy@erdogan.edu.tr; 2 Anesthesiology Department of Sadi Konuk Training and Research Hospital, 34147 Istanbul, Turkey; imrakici@hotmail.com; 3 Department of Pulmonology, Faculty of Medicine, Recep Tayyip Erdogan University, 53020 Rize, Türkiye; aziz.gumus@erdogan.edu.tr; 4 Department of İnternale Medicine, Faculty of Medicine, Recep Tayyip Erdogan University, 53020 Rize, Türkiye; kamil.konur@erdogan.edu.tr

**Keywords:** gastrointestinal bleeding, perfusion, fluid deficit, perfusion index, pleth variability index

## Abstract

**Background:** This study aimed to investigate the potential benefits of the Perfusion Index (PI) and Pleth Variability Index (PVI), which indicate tissue microcirculatory perfusion, in the management of fluid therapy in patients with gastrointestinal (GI) bleeding, as well as their relationship with laboratory parameters. **Methods:** PI, PVI, and oxygen saturation values were measured using a Masimo pulse oximeter, along with routine tests, in patients with overt gastrointestinal bleeding presenting with melena, hematemesis, or hematochezia. These values were reassessed in patients who received treatment and fluid replacement prior to discharge. Baseline and discharge values were compared. The study examined whether there was a significant change in these values following treatment. The relationship between PI and PVI values and demographic findings, oxygen saturation, urea, creatinine, hematocrit, hemoglobin, and white blood cell counts were evaluated. **Results:** A total of 100 patients were included in the study, 55 (55%) of whom were male and 45 (45%) female, with a mean age of 65.8 ± 8.3 years. Of the patients, 81 had upper GI bleeding, and 19 had lower GI bleeding. Of the upper GI bleeds, 6 were esophageal variceal bleeds, and 75 were due to other causes. Perfusion index (PI) and plethysmographic variability index (PVI) measurements were obtained at initial admission and prior to discharge. The discharge PI value increased significantly compared with the admission PI value, whereas the discharge PVI value decreased significantly compared with the admission PVI value. PVI value. (*p* < 0.001) The relationship between PI and PVI values and other variables was analyzed using Spearman’s correlation analysis. A significant negative correlation was found between PI-admission and PVI-admission, and between PI-discharge and PVI-discharge. (*p* < 0.001). Additionally, a significant but mild positive correlation was observed between PVI-out and age, creatinine, and urea. (*p* < 0.010, *p* < 0.030) Our results demonstrated a significant difference between PI-in and PI-out values following fluid replacement therapy. PVI-in and PVI-out values also differed significantly. There was a significant negative correlation between PI and PVI values. These findings suggest that the effectiveness of fluid replacement therapy can be monitored using PI and PVI. **Conclusions:** Monitoring fluid therapy in cases of gastrointestinal bleeding using the Massimo pulse oximeter is a simple, inexpensive, repeatable, noninvasive, feasible and appropriate method. Our study has significantly demonstrated that fluid replacement leads to an increase in PI values and a decrease in PVI values.

## 1. Introduction

The perfusion index (PI) is a parameter that reflects microcirculation and peripheral perfusion. The peripheral effects of macrovascular events can also affect microvascular circulation. Therefore, it provides information about the body’s fluid balance. Fluid therapy administered in inappropriate volumes can be detrimental to patients. Consequently, accurate determination of fluid requirements is crucial. While appropriate fluid therapy can improve prognosis, both insufficient and excessive fluid administration can worsen outcomes. Central venous oxygen saturation, an invasive measure reflecting macrovascular circulation, does not necessarily indicate adequate microvascular perfusion, even when within normal limits. Therefore, a non-invasive test that reflects microcirculatory perfusion plays a crucial role in daily clinical practice. The Perfusion Index (PI) was developed to address this need.

PI is defined as the ratio of the pulsatile signal (PD), derived primarily from arterial blood flow, to the non-pulsatile signal (POD), derived from venous and other non-pulsatile tissues, and is calculated as: PI = (PD/POD) × 100%. PI can be measured using the Massimo pulse oximeter (Massimo Corp., Irvine, CA, USA) includes a module that monitors respiratory changes in the plethysmographic waveform derived from the perfusion index (PI). The pleth variability index (PVI), which measures the PI’s response to respiratory changes is also displayed. PVI reflects the variation in PI during the respiratory cycle. PVI is calculated as (PImax − PImin)/PImax × 100% [1]. Its non-invasive, simple, inexpensive, and reproducible nature offers significant advantages. PI is not expressed in absolute units; rather, it is a ratio. Since it is affected by movement, measurements should be obtained while the patient is stationary. Normal values in healthy individuals vary among studies; one study reported a mean value of 3.9 (2.9–6.1). Other new methods for measuring microcirculatory perfusion have been developed. These include sublingual microcirculation, tissue oxygen saturation, and transcutaneous partial oxygen pressure using side-flow dark-field (SDF) imaging [1,2,3]. However, none of these methods can be measured easily or inexpensively compared to the perfusion index. PI is influenced by cardiac output, blood volume, vascular tone, microcirculatory status, and perfusion pressure, as well as by age, gender, pain, peripheral vascular disease, mechanical ventilation, and body temperature. PI can be measured at different sites, such as the fingers, forehead, and earlobe, the most practical measurement is obtained from the middle finger. Although measurements may vary between different sites or across repeated assessments at the same site, PI remains a valuable tool, particularly in intensive care units and bedside settings. Its noninvasive nature and reproducibility provide significant convenience. PI is very low in critical illness conditions.

A very low PI is associated with increased mortality in patients with septic or hypovolemic shock, following cardiac arrest and during mechanical ventilation. It has also been demonstrated that the PI serves as a parameter indicating the severity of the condition and the prognosis [4]. Fluid resuscitation guided by the perfusion index (PI) was shown to be more effective than lactate-guided resuscitation with respect to organ dysfunction [5]. Moreover, the integration of PI with macrocirculatory indicators, such as central venous oxygen saturation (ScvO_2_), may further enhance the effectiveness of fluid management [6]. PI can also provide helpful information in weaning the patient from mechanical ventilation, distinguishing ST-elevation myocardial infarction following cardiac arrest, determining treatment options in pulmonary embolism, and assessing the need for transfusion in gastrointestinal bleeding [1,2,3,4,5,6]. The perfusion index (PI) may be a valuable tool for guiding norepinephrine dose titration [7]. In patients receiving renal replacement therapy (hemodialysis), a low PI may reflect hypovolemia, while PI may also assist in determining the optimal volume of fluid removal in patients with hypervolemia [8,9]. Painful stimuli induce sympathetic activation, leading to increased peripheral vascular tone and a consequent reduction in the perfusion index (PI). Accordingly, PI may be used as an objective surrogate marker for pain assessment in unconscious or non-communicative patients [10]. PI has been used as an early indicator of regional anesthesia success by detecting vasodilation associated with sympathetic tone inhibition through an increase in PI. Additionally, a PI value greater than 3.5% prior to spinal anesthesia has been identified as a risk factor for anesthesia-induced hypotension. It may also cause inaccuracies in capillary blood glucose measurements and oxygen saturation readings.

PVI is similarly affected by the same factors that influence PI. Its reliability is low in cases of impaired peripheral perfusion. The normal range is accepted as 7–20%. Higher values are considered pathological. PVI serves as an indicator of fluid deficit and impaired peripheral perfusion [3,11].

## 2. Materials and Methods

**Figure 1:**
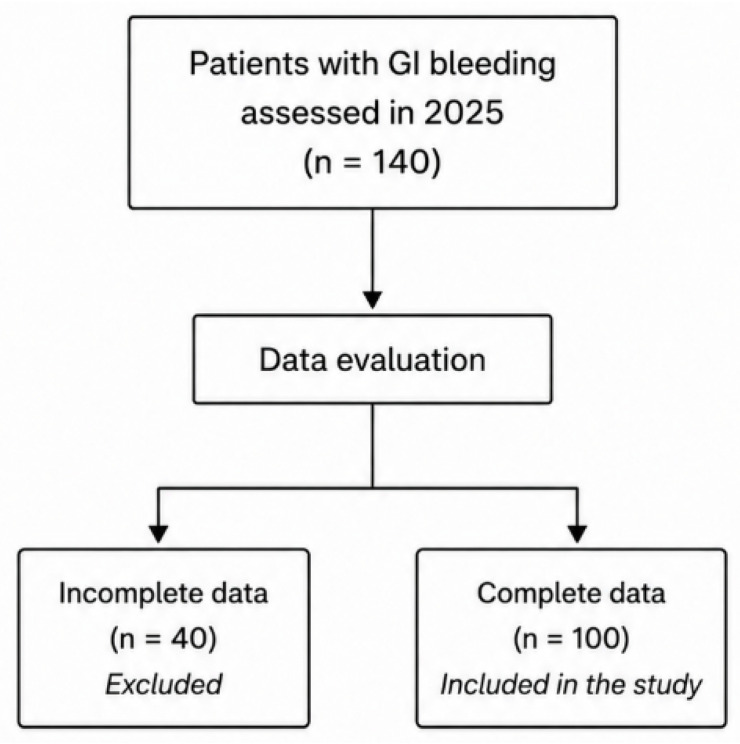
Study design.

A total of 140 patients with gastrointestinal bleeding were followed in 2015. Of these, 100 patients with complete data were included in the study (Figure 1).

Ethical approval was obtained from the Recep Tayyip Erdoğan University Non-Interventional Clinical Research Ethics Committee under number E-40465587-050.01.04-1333 and 2025/24. Our study is a prospective observational study. Patients were admitted due to overt GI bleeding, such as melena, hematemesis, or hematochezia. On the day of admission, patients underwent a complete blood count, biochemical tests, and measurements of oxygen saturation, PI, and PVI using a Masimo pulse oximeter (Massimo Corp. Irvine, CA, USA). Patients received daily fluid or blood transfusions ranging from one to three units (average 2 L). Fluid replacement was adjusted based on PI and PVI values. PI and PVI values were re-evaluated at the time of discharge. Patients with melena, hematemesis, and hematochezia were included in the study regardless of etiology. However, patients admitted due to iron deficiency anemia or chronic anemia and outpatients, were excluded from the study. Additionally, patients for whom we could not measure PVI while measuring PI using a pulse oximeter were excluded from the study. The aim was to assess whether there were changes in PI and PVI values before and after fluid replacement and to examine their relationship with hematocrit, hemoglobin, white blood cell count, oxygen saturation, creatinine, and urea levels. Fluid replacement therapy consisted of isotonic sodium chloride, 5% dextrose, lactated Ringer’s solution, and blood transfusion for some patients. All patients routinely received 1000 mL of isotonic saline as maintenance intravenous fluid therapy. When oral intake was withheld, an additional 1000 mL of either 5% dextrose solution or lactated Ringer’s solution was administered. Patients with additional fluid requirements received a further 1000 mL of isotonic saline. Red blood cell transfusion was indicated for patients with a hemoglobin level < 7 g/dL. In patients with ischemic heart disease, a more restrictive transfusion threshold was not applied, and the hemoglobin level was maintained at >9 g/dL. All patients were hemodynamically stable and were managed in the general ward, with no patient requiring admission to the intensive care unit. Patients were assessed and monitored using the Clinical Rockall Score. Upper gastrointestinal endoscopy was performed within the first 24 h of admission in patients who had completed at least 8 h of fasting and remained hemodynamically stable. All patients were successfully treated with medical and endoscopic therapy, and no patient required angiographic or surgical intervention.

Statistical analyses were performed using IBM-SPSS software (SPSS version 21; SPSS Inc., Chicago, IL, USA). The study was conducted in a region with a population of approximately 250,000. Based on the annual incidence of upper gastrointestinal bleeding, the required sample size was calculated to be approximately 100 patients using a 95% confidence level and a 5% margin of error. A post hoc power analysis demonstrated that the study achieved a statistical power of 100%. The normality of continuous variables was assessed using the Kolmogorov–Smirnov test. Continuous variables were reported as mean ± standard deviation and median (interquartile range), while categorical variables were reported as percentages. The Wilcoxon method was used to compare dependent variables. The relationship between variables was examined using Spearman’s correlation method. A *p*-value of <0.05 was considered statistically significant.

## 3. Results

A total of 100 patients, including 55 (55%) men and 45 (45%) women with a mean age of 65.8 ± 8.3, were included in the study with a diagnosis of upper and lower GI bleeding. A complete blood count (CBC) and renal function tests (RFTs) were performed for all patients upon admission. Additionally, oxygen saturation levels were recorded. The patients’ demographic characteristics, RFT results, complete blood count values, and oxygen saturation levels are presented in Table 1. Of these patients, 81 had upper GI bleeding, and 19 had lower GI bleeding. Among the upper GI bleeds, 6 were due to esophageal variceal bleeding, and 75 were due to other caus.

**Figure 2:**
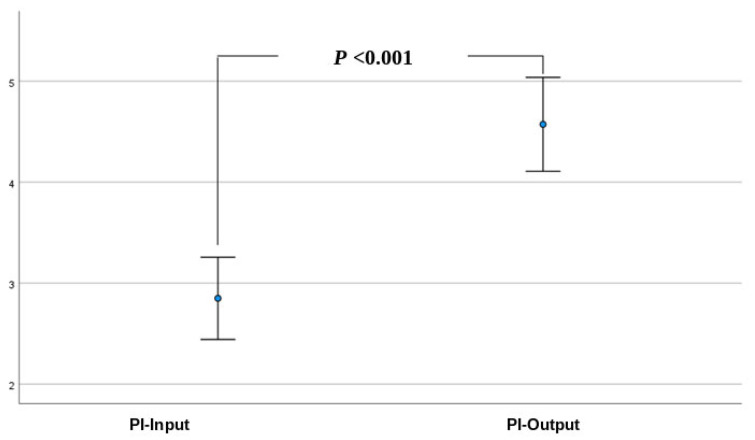
Error bar graph showing the difference between the PI input and output values.

**Figure 3:**
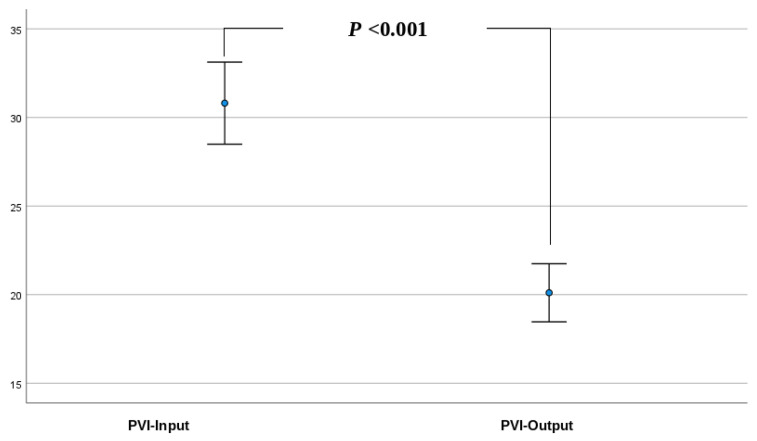
Error bar chart showing the difference between PVI input and output values.

Perfusion index (PI) and pleth variability index (PVI) measurements were recorded at initial admission and before discharge. The discharge PI value increased significantly compared with the admission value, whereas the discharge PVI value decreased significantly (Table 2, Figure 2 and Figure 3).

The relationship between PI and PVI values and other variables was examined using Spearman’s correlation analysis. A moderately significant negative correlation was found between PI-in and PVI-in, and between PI-out and PVI-out. Although correlation analysis demonstrated weak associations between discharge PVI and age, urea, and creatinine, these associations were not significant in the multivariable regression analysis. weak positive correlation was observed between PVI-out and age, creatinine, and urea (Table 3 and Table 4).

**Figure 4:**
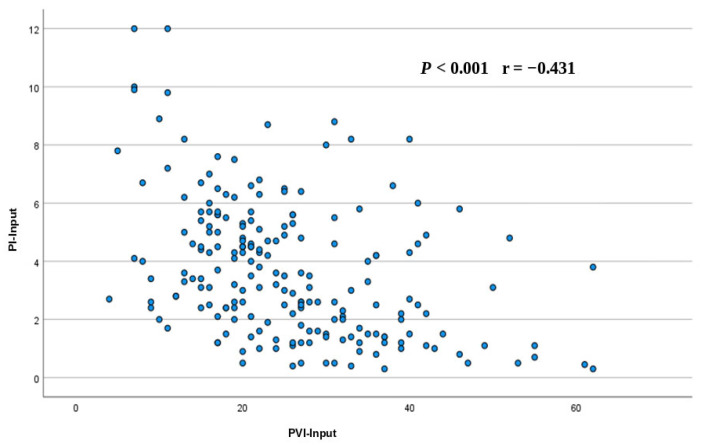
Illustration of the negative correlation between PI-input and PVI-input using a scatter plot.

**Figure 5:**
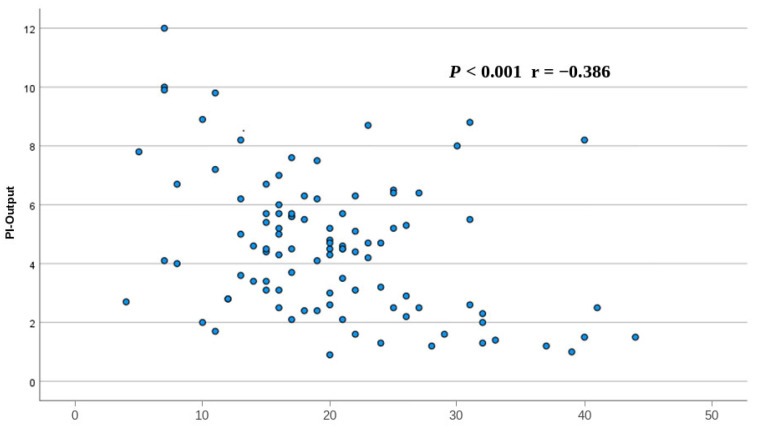
Scatterplot illustrating the negative correlation between PI output and PVI output.

Subgroup analysis showed no statistically significant differences in admission or discharge PI and PVI values between patients with upper and lower gastrointestinal bleeding (Table 5, Figure 4 and Figure 5).

## 4. Discussion

PI and PVI are parameters that reflect peripheral microcirculation and perfusion. These parameters can be easily and conveniently measured using a pulse oximeter. PI is defined as the ratio of the pulsatile signal (PD), derived primarily from arterial blood flow, to the non-pulsatile signal (POD), derived from venous and other non-pulsatile tissues. PVI is a parameter that reflects the respiratory variability of PI. Both indices have been studied in a wide variety of diseases regarding their applicability in fluid replacement therapy. They are particularly used for perioperative monitoring in intensive care units and anesthesiology practice [4,5,6,7]. Their efficacy has been evaluated in cases of septic or hypovolemic shock, during major cardiovascular surgery, in patients on mechanical ventilation, and following cardiac arrest. They are also used in pediatric patient groups and neonatal intensive care units [8,9,10,11,12,13]. The Massimo pulse oximeter, which is also used for measurements, is designed for this purpose and displays pulse rate, oxygen saturation, PI, and PVI values on the same screen. Although measurements can be taken from the fingers, forehead, and earlobe, the most suitable measurement site is the middle finger [2,3,14,15]. Measurement from a larger surface area enhances its effectiveness. Although it is easy, inexpensive, repeatable, and non-invasive, its limited use in daily practice is attributed to measurement heterogeneity and the inability to establish an objective cutoff value. Different cutoff values have been reported in various studies [15]. PI values also vary depending on body position and movement. Some studies have reported that a PI value below 1.3 is associated with a poor prognosis and increased mortality [14,15,16]. Since the PVI value is a variant of the PI, it can similarly be used to monitor fluid replacement therapy. However, it is more significantly affected by cardiopulmonary exercise. In our study, low PI and elevated PVI values changed significantly after fluid replacement, with an increase in PI and a decrease in PVI. In our study, the goal in determining the fluid replacement volume was to bring the PI and PVI values within the normal range. However, the PI and PVI values of some patients were normal at admission. The fluid requirements for these patients were managed according to routine protocols for patients with GI bleeding. Nevertheless, changes in PI and PVI values were also observed in these patients [17,18,19,20]. On the other hand, there is a study reporting a decrease in PI values after blood donation in blood donors [17]. In our study, the PVI value also showed an inverse correlation with the PI value. The PVI cutoff value has been accepted within a wide range of 7–20%. In our study, the highest PVI value was 62. While this value decreased with fluid replacement, it did not return to normal levels in the majority of patients. Another finding observed in our study is that the Massimo pulse oximeter does not display the PVI value, particularly in elderly patients and those with low PI. Such patients were excluded from the study. A study in the literature evaluating the effect of PI in upper GI bleeding reported that the need for red blood cell transfusion increases when the PI value is below 1.17, and mortality increases significantly when it is below [17,18,19,20]. In our study, two (2%) patients were lost to follow-up over one year. In our study, no correlation was found between hematocrit, hemoglobin, white blood cell count, oxygen saturation, urea, and creatinine levels and the PI and PVI values. No evaluation was performed regarding mortality. Crystalloid solutions and blood transfusions were administered for fluid replacement. Since the type of fluid was not the subject of this study, no separate evaluation was conducted. The literature reports that colloid solutions result in a higher PI value compared to crystalloid solutions [21].

Another important point is that a high PI value may result from vasodilation caused by local factors (heat, pain, local anesthesia). This condition may serve as a warning sign of hypotension that could occur following spinal anesthesia [22,23].

Previous studies have demonstrated that the perfusion index (PI) and pleth variability index (PVI) are valuable tools for monitoring hemodynamic status in neonates admitted to neonatal intensive care units after either cesarean section or vaginal delivery [24,25,26]. Furthermore, these indices have been reported to be effective in guiding fluid resuscitation, assessing hemodynamic status, and predicting mortality in patients undergoing major cardiac surgery and in those with cardiac arrest [27].

Our study has some limitations. First of all, measurements were taken by different physicians from different fingers. This may have introduced heterogeneity. Secondly, there is no consensus on the average value of PI and PVI because their cutoff values vary across different studies. Third, there is no established measure indicating the optimal amount of fluid to administer or the target PI value in patients with gastrointestinal (GI) bleeding who have normal PI values. Fourth, the effects of the different fluids administered on increasing PI or decreasing PVI were beyond the scope of this study.

Overall, our findings suggest that PI is a promising non-invasive bedside parameter for monitoring fluid resuscitation response in patients with gastrointestinal bleeding. Further large-scale prospective multicenter studies are needed to establish standardized PI and PVI cutoff values for guiding fluid therapy and to evaluate whether PI-guided fluid therapy improves clinical outcomes, including transfusion requirements, intensive care admission, and mortality.

## 5. Conclusions

The use of the Masimo pulse oximeter, which provides PI and PVI measurements, is a practical, simple, inexpensive, and reproducible method for guiding fluid replacement therapy in patients with gastrointestinal (GI) bleeding. Our study results demonstrate that PI increases with fluid resuscitation, while PVI decreases. These findings may help guide clinical management.

## Figures and Tables

**Table 1:** Demographic and Biochemical Characteristics of Patients.

Gender	
Male	55 (55%)
Female	45 (45%)
Age	65.8 ± 8.3
Complete blood count	
Hb	8.76 ± 1.49
Hct	26.6 ± 4.3
White Blood Cells	6647 ± 1146
Renal Function Tests	
Urea	56 (45–74)
Creatinine	0.90 (0.70–1.17)
Oxygen saturation	95.5 ± 2.5

Data are presented as count (%), mean ± standard deviation, and median (interquartile range).

**Table 2:** Demonstration of the difference between PI and PVI values measured at admission and discharge.

Variables	Admission	Discharge	*p* Value
PI	2.45 (1.20–4.20)	4.50 (2.60–5.92)	<0.001
PVI	28.5 (22.0–37.0)	19.5 (15.0–24.8)	<0.001

Data are presented as median (interquartile range).

**Table 3:** Demonstration of the relationship between variables using Spearman’s correlation analysis.

	PVI-Output	PI-Output	PVI-Input	PI-Input
PI-input	*p* < 0.001 r: −0.340	*p < 0.001* r: 0.830	*p* < 0.001 r: −0.431	
PVI-input	*p* < 0.001 r: 0.804	*p*: 0.078 r: −0.177		
PI-output	*p* < 0.001 r: −0.386			-
Age	*p*: 0.003 r: 0.295	*p*: 0.179 r: −0.135	*p*: 0.009 r: 0.260	*p*: 0.412 r: −0.083
Creatinine	*p*: 0.010 r: 0.256	*p:* 0.139 r: −0.149	*p*: 0.236 r: 0.120	*p*: 0.272 r: −0.111
Urea	*p*: 0.034 r: 0.213	*p*: 0.264 r: −0.113	*p*: 0.340 r: 0.096	*p*: 0.177 r: −0.136
Hb	*p*: 0.767 r: 0.030	*p*: 0.742 r: −0.033	*p*: 0.871 r: −0.016	*p*: 0.470 r: −0.073
Hct	*p*: 0.684 r: 0.041	*p*: 0.794 r: −0.026	*p*: 0.979 r: −0.003	*p*: 0.647 r: −0.046
So2	*p*: 0.149 r: −0.145	*p*: 0.562 r: −0.059	*p*: 0.211 r: −0.126	*p*: 0.382 r: −0.088

**Table 4:** Multiple Linear Regression Analysis of the Association Between Discharge PVI Values and Age, Urea, and Creatinine.

	B	Std. Error	Beta	*p* Value
Constant	4.995	6.534		0.446
Age	0.203	0.105	0.204	0.057
Urea	0.001	0.024	0.051	0.960
Creatinine	1.585	1.062	0.160	0.139

**Table 5:** Comparison of Perfusion Index (PI) and Pleth Variability Index (PVI) Values Measured at Admission and Discharge in Patients with Upper and Lower Gastrointestinal Bleeding.

	Patients with Upper Gastrointestinal Bleeding (n: 81)			Patients with Lower Gastrointestinal Bleeding (n: 19)		
	Admission	Discharge	*p* Value	Admission	Discharge	*p* Value
PI	2.4 (1.2–4.3)	4.5 (2.6–6.2)	<0.001	2.5 (1.1–3.8)	4.5 (2.3–5.2)	<0.001
PVI	28 (23–39)	19 (15–24)	<0.001	30 (20–37)	20 (8–26)	<0.001

## Data Availability

The original contributions presented in this study are included in the article. Further inquiries can be directed to the corresponding author.
